# Effects of Life Table Models on the Evaluation of Excess Mortality

**Published:** 2010-09

**Authors:** Mahmood Sheikh Fathollahi, Mahmood Mahmoodi, Kazem Mohammad, Hojjat Zeraati

**Affiliations:** *Department of Epidemiology and Biostatistics, School of Public Health, Tehran University of Medical Sciences, Tehran, Iran*

**Keywords:** excess mortality, relative survival, life table models, coale-demeny patterns, mazandaran, gastrointestinal tract cancer

## Abstract

**Background:**

Northern regions of Iran have been encountered to dominate malignancies of gastrointestinal (GI) tract. We aimed to examine the excess mortality due to the GI tract cancer in Mazandaran province.

**Methods:**

Socio-demographic and clinical data of 484 patients with GI cancer collected during the years 1990-1991 were available from Babol Cancer Registry Center in Iran. Patients were followed-up for a maximum period of 15 years by the year 2006. The Coale-Demeny life tables were established for each combination of birth cohort and sex of patients, and were considered as the reference population in estimating excess mortality rates. The relative and additive mortality models for excess mortality estimation were used.

**Results:**

The sample of subjects encompassed 66.3% men and 33.7% women, with mean age 58.26 ± 10.90 years. Esophageal cancer appeared to be the most common one, and endoscopy was the general method for cancer detection. Survival rate in 15 years following diagnosis was nearly 6%. Excess mortality estimated by each of the relative and additive models reached the most value in the first two years of observation in both genders and according to each of the Coale-Demeny regional patterns.

**Conclusion:**

considering individuals in a population come from different cohorts with different mortality patterns, it might be recommended to construct distinct life tables for different birth cohorts when estimating excess hazard. The West model as a general pattern is recommended to represent mortality patterns in countries whose registration systems either do not exist or are so affected by omission and other errors.

## INTRODUCTION

Cancer is estimated to become the leading cause of death in many countries of the world in 2010. Nearly 28 million people worldwide are affected by some sort of the disease (1). Gastrointestinal (GI) tract cancers together consisted of the most common broad group of cancers in 2007 (2). Annually around 50,000 new cases of cancer occur across the 70.4 million population of Iran, in which GI tract is reported as the most general organ system involved with over 38% of all cancers. In addition, Stomach, Esophageal, and Colorectal cancers are the three most common cancers among Iranian males; however, Iranian females convey the highest rates of cancers of Esophagus, Stomach, and Colorectal after breast cancer (3, 4).

Excess mortality is a statistical method frequently used in population-based studies to evaluate the effect of a particular disease on mortality especially when the cause of death is known unreliable or unavailable (5, 6). When modeling mortality in clinical or epidemiologic studies, it is common to use the mortality rate of the general population as a standard reference for comparisons. Namely, one usually relies on the published life tables as the reference population to compare the mortality experience of a group of diseases to a known population (7, 8). A major limitation of most of these life tables is that individuals in a population basically come from different cohorts with different mortality experiences, while information of mortality rates of different cohorts is as if pooled and combined into a single table. This disparity in the pattern of mortality across cohorts can severely affect life table figures and therefore excess mortality measures, which necessitates an adequate adjustment for birth cohort effect during the process of establishing life tables.

Unfortunately, in the majority of developing countries including Iran, registration systems either do not exist or are so affected by omission and other errors. Indeed, there may be little known on the actual age pattern of mortality in these populations, so as measures based on the data that they produce fail to reflect properly either levels or trends of mortality. A number of model life table systems have been developed for use in such cases, but the two most commonly used are the Coale-Demeny and the United Nations Model Life Tables for Developing Countries (9-11).

We came to examine long-term excess mortality due to the GI tract cancer in Mazandaran, the province with the dominating rate of GI tract cancer. For this purpose, Coale-Demeny model life tables were constructed for each combination of birth cohort and sex of patients and they were considered as the basis for estimating excess mortality. Data from Babol cancer registry system during the years 1990 and 1991 was studied.

## MATERIAL AND METHODS

### Babol Cancer Registration

The Caspian Cancer Registry established in 1969 by joint efforts of the Institute of Public Health Research of Tehran University and the International Agency for Research on Cancer (IARC), in the city of Babol in Mazandaran province. The city is located approximately 20 kilometers south of Caspian Sea on the west bank of Babol Rood River. As a population-based cancer registry, it has provided a reliable source of data on cancer incidence in the Caspian littoral of Iran (12). These efforts were, however, interrupted by the Revolution of Iran and the subsequent socio-political events of the 1980s, but it recommenced the regular activities again in the year 1990 as a local cancer registry under the supervision of Tehran University of Medical Sciences. The data sources were mainly reports collected from pathology laboratories, hospitals, and radiology clinics offering samples with cancer progression. The coding of the samples was done under the direct supervision of pathology specialists based on the international classification of disease for oncology (ICD-O) coding (13).

A total of 484 patients diagnosed with GI tract cancer were registered at the Babol cancer registration in Mazandaran province between the years 1990-1991 entered into the study. The collected sample contained 359 cases with esophageal, 110 with stomach, and 15 with colorectal cancers. Patients were followed up for a maximum period of 15 years by the year 2006. The socio-demographic and clinical data were obtained through a structured questionnaire and the patients’ clinical records. The data available for analysis included the following: gender, age at the time of diagnosis, current job, education, ethnicity, place of residence, pathologic diagnosis, and diagnostic methods. The study was approved by the ethics committee of Tehran University of Medical Sciences.

### Creating Life Table Models

Since in many countries including Iran, death registration is incomplete or nonexistent, accurate life tables cannot be established from the data available. Model life tables have been developed for use in such cases. The Coale-Demeny model life tables are amongst the most commonly used models and consist of four sets or models, each representing a distinct mortality patterns, including North, South, East, and West (11). By having in hand the measure of Infant Mortality Rate (IMR) for each *year of birth*, defined as the number of newborns dying under a year of age divided by the number of live births that year, Coale-Demeny model life tables can be constructed. Concerning the Mazandaran province, IMR was available for birth years after 1965; therefore, linear extrapolation methods were invoked to approximate IMR for birth years before 1965.

Because the study patients came from different cohorts with experiencing different mortality patterns, men were classified into *five* distinct cohorts of 1911-1920, 1921-1930, 1931-1940, 1941-1950, and 1951-1961, and women into *four* cohorts of 1921-1930, 1931-1940, 1941-1950, and 1951-1961. It should be noted that an average IMR was calculated for each combination of birth cohort and sex of patients (Table 1).

**Table 1:** Average infant mortality rate (IMR) per thousand separated by gender and birth cohort (Total n=484)

Gender / Birth cohort	Average IMR per thousand

Male gender	
1911-1920 (n=6)	383
1921-1930 (n=31)	324
1931-1940 (n=97)	270
1941-1950 (n=107)	231
1951-1961 (n=79)	185
Female gender	
1921-1930 (n=12)	333
1931-1940 (n=34)	285
1941-1950 (n=51)	240
1951-1961 (n=66)	179

The MORTPAK software package version 4.0 for Windows (the United Nations Software Package for Demographic Measurement in Developing Countries) as the first version with a Windows user interface, developed by the United Nations Population Division Department of Economic and Social Affairs in 1988, was applied to construct the Coale-Demeny model life tables of each birth cohort separated by gender. MORTPAK includes 17 applications in the areas of population projection, life-table construction, and other procedures.

The application MATCH generates model life tables corresponding to given levels of mortality. By specifying the model pattern (any of the four Coale-Demeny patterns) and the sex category, and the measure of IMR of the corresponding cohort, the Coale-Demeny model life tables were established. Thus, a number of *five* life tables for men and *four* for women corresponding to each birth cohort were constructed separately for each family of Coale-Demeny models. As one step further, we also employed the UNABR procedure to graduate a set of age-specific mortality rates in traditional age groupings of 0-1, 1-5, 10-15, ..., produced by the MATCH procedure in the former step, into single year values of dying and survivors. Indeed, the unabridged life table output was presented for single years of age 0-100. Thus, these established life tables were in fact considered as the standard reference population life tables adjusted for birth cohort effect and sex of patients.

### Computing Excess Mortality

The term excess mortality is used to describe the additional hazard rate in a study population relative to that seen in a reference population with the same age, sex, and where applicable, race distributions. A simple model is introduced by Andersen and Væth in 1989 and a nonparametric estimate of the integrated excess mortality rate is also given (14).

The first model for excess mortality considers the effect of the disease on mortality multiplicative and is widely known as the *relative mortality model*. The model is defined as

*h*_j_(*t*)=*β*(*t*).*θ*_j_(*t*), *j=1,2,…,n*.

Here, *h*_j_(*t*) is the hazard rate at time t for the *j^th^* patient under study, *β*(*t*) is the *multiplicative mortality*, and *θ*_j_(*t*) is the reference (population) hazard rate for the individual.

Direct estimation of *β*(*t*) is difficult. Instead, B(*t*), the *cumulative relative mortality*, can be estimated from the data at hand. A crude estimator of the relative risk function β(*t*) is given by the slope of the estimated cumulative relative mortality estimator (15).

The second model comparing the study population to a reference one is known as the *excess or additive mortality model*. The model can be described by

*h*_j_(*t*)=*α*(*t*)+*θ*_j_(*t*), *j=1,2,…,n*

in which *h*_j_(*t*) and *θ*_j_(*t*) are defined as before, and *α*(*t*) is the *additive mortality*.

Again, direct estimation of *α*(*t*) is difficult. Instead, *A*(*t*), the *cumulative excess mortality* is estimated from the data. A crude estimator of the additive risk function *α*(*t*) is given by the slope of the estimated cumulative excess mortality estimator (15).

Note that in the two proposed models for excess mortality the established life tables adjusted for birth cohort effect and sex of patients were considered as the standard reference population and *θ*_j_(*t*) was replaced by the appropriate values of the hazard rate column of the tables.

## RESULTS

The resultant sample was comprised of 484 GI tract cancer patients, out of which 66.3% were male and 33.7% were female. The mean age of the patients was 58.26 ± 10.90 (mean ± SD) years (range 40 to 90 yr).

Table 2 shows a summary of socio-demographic and clinical data for the cancerous patients. As can be seen in the table, esophageal cancer is the most common cancer and stomach is the second most frequent one and colorectal cancer accounts for just over 3% of all the sorts of GI tract cancers. Furthermore, esophageal cancer was more prevalent in women while stomach cancer was found more frequently in men. Nearly 85% of all cancers were detected by the direct endoscopy and biopsy, and the distribution of cancer detection methods was not statistically different across the two genders (Table 2).

**Table 2:** Characteristics of patients with GI tract cancer diagnosed*

Characteristics	Total (n=484)	Male (n=321)	Female (n=163)	*P*-value

Age (years)	58.26±10.90	59.55±10.56	55.72±11.15	<0.001
Place of residence				0.231
Rural	256 (52.9)	176 (54.8)	80 (49.1)	
Urban	228 (47.1)	145 (45.2)	83 (50.9)	
Province				0.117
Mazandaran	288 (59.5)	199 (62.0)	89 (54.6)	
Golestan†	196 (40.5)	122 (38.0)	74 (45.4)	
Type of cancer				0.011
Esophageal	359 (74.2)	225 (70.1)	134 (82.2)	
Stomach	110 (22.7)	83 (25.9)	27 (16.6)	
Colorectal	15 (3.1)	13 (4.0)	2 (1.2)	
Method of cancer detection				0.713
Clinical diagnosis	35 (7.2)	21 (6.5)	14 (8.6)	
Direct endoscopy and biopsy	410 (84.7)	274 (85.4)	136 (83.4)	
Conventional chest x-ray	39 (8.1)	26 (8.1)	13 (8.0)	
Family history of cancer	142 (29.3)	88 (27.4)	54 (33.1)	0.192
Education				<0.001
Literate	52 (10.7)	46 (14.3)	6 (3.7)	
Illiterate	432 (89.3)	275 (85.7)	157 (96.3)	
Job				<0.001
Farmer	252 (52.1)	230 (71.7)	22 (13.5)	
Worker	44 (9.1)	44 (13.7)	0	
Employee	7 (1.4)	6 (1.9)	1 (0.6)	
Housewife	135 (27.9)	0	135 (82.8)	
Others	46 (9.5)	41 (12.8)	5 (3.1)	
Marital status				0.004
Married	459 (94.8)	311 (96.9)	148 (90.8)	
Single	25 (5.2)	10 (3.1)	15 (9.2)	
Cigarette smoking	215 (44.4)	185 (57.6)	30 (18.4)	<0.001
Ethnicity				0.983
Aryan	327 (67.6)	216 (67.3)	111 (68.1)	
Torkaman	100 (20.7)	67 (20.9)	33 (20.2)	
Others	57 (11.8)	38 (11.8)	19 (11.7)	

* Data are presented as mean ± SD or n (%);

† This new province was created as a result of the division of Mazandaran province into two smaller administrative units in the year 1997; GI, Gastrointestinal.

**Figure 1:**
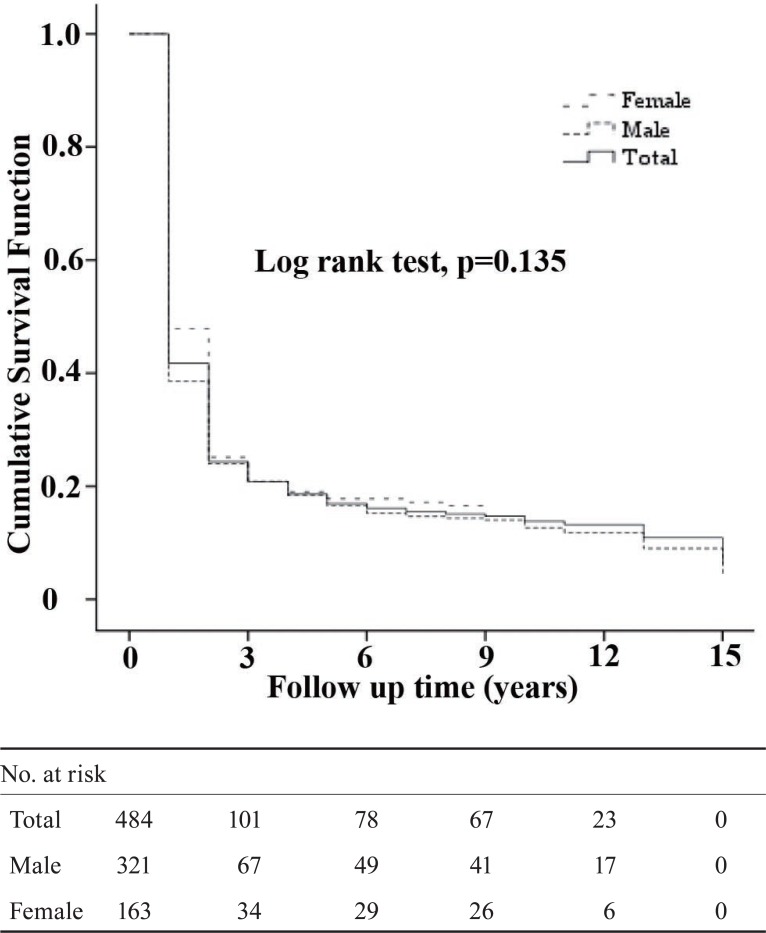
Kaplan–Meier plot of cumulative survival of mortality to fifteen years after diagnosis for the gastrointestinal tract cancer study in Mazandaran province in Iran.

Follow-up time ranged from 1 to 15 years with an average follow-up of 3.09 ± 3.82 years in which men were 3.02 ± 3.82 and women 3.22 ± 3.84 years on study. During the period under study, 88.0% (n = 426) mortality events occurred that men accounted for over two third (67.8%) and women for 32.2% of deaths. The Kaplan-Meier method of survival analysis estimated that the survival rates in 5, 10, and 15 years following diagnosis were 16.9%, 13.8%, and 6.2%, respectively. The overall patient survival rate was not statistically different between the two genders (Figure 1).

Table 3 shows the estimated excess mortality based on the four Coale-Demeny regional model life tables. According to the relative mortality model and using the East model life table, in the first two years of observation, cancerous patients were 30-40 times more likely to die than comparable individuals in the reference population. In years 3-10, the patients were between 2-6 times, and 11-15 years following diagnosis they were 3-4 times more likely to die. On the other hand, according to the additive mortality model and the same life table pattern, we see that a crude estimate for the excess number of deaths, given by the slope of the estimated cumulative excess mortality, is a function which is about 0.20 for the first two years of observation, declines to 0.05 during 3-10 years, and then increases to 0.10 afterwards (Table 3). For example, after 10 years on study, the cumulative excess mortality was estimated to be 0.53 (a crude 95% CI, 0.41-0.65) according to the East life table, which shows that in a group of 100 patients, we might see 53 more deaths after 10 years of GI tract cancer diagnosis than we would expect to see in the reference population. Similarly, the cumulative excess mortality was estimated to be 0.36, 0.33, and 0.42 after 10 years following diagnosis when the North, South, and West models, respectively, were used to estimate excess mortality (data not shown).

**Table 3:** Estimated excess mortality using relative and additive mortality models and according to Coale-Demeny regional life table models for 484 patients with GI tract cancer diagnosed

Excess mortality model	Coale-Demeny life table model	0-2 years after diagnosis	3-10 years after diagnosis	11-15 years after diagnosis

Relative^a^	North	20-30 times	1-5 times	2-3 times
	South	20-30 times	1-5 times	2-3 times
	East	30-40 times	2-6 times	3-4 times
	West	25-35 times	2-5 times	2-4 times
Additive^b^	North	0.10 slope	0.03 slope	0.06 slope
	South	0.10 slope	0.03 slope	0.05 slope
	East	0.20 slope	0.05 slope	0.10 slope
	West	0.15 slope	0.04 slope	0.09 slope

a h_j_(t)=β(t).θ_j_(t), j=1,2,...,n. Here, h_j_(t) is the hazard rate at time t for the j^th^ patient under study, β(t) is the relative mortality, and θ_j_(t) is the reference (population) hazard rate for the individual;

b h_j_(t)=α(t)+θ_j_(t), j=1,2,...,n. Here, h_j_(t) and θ_j_(t) are defined as before, and α(t) is considered as the additive mortality; GI, Gastrointestinal.

**Figure 2:**
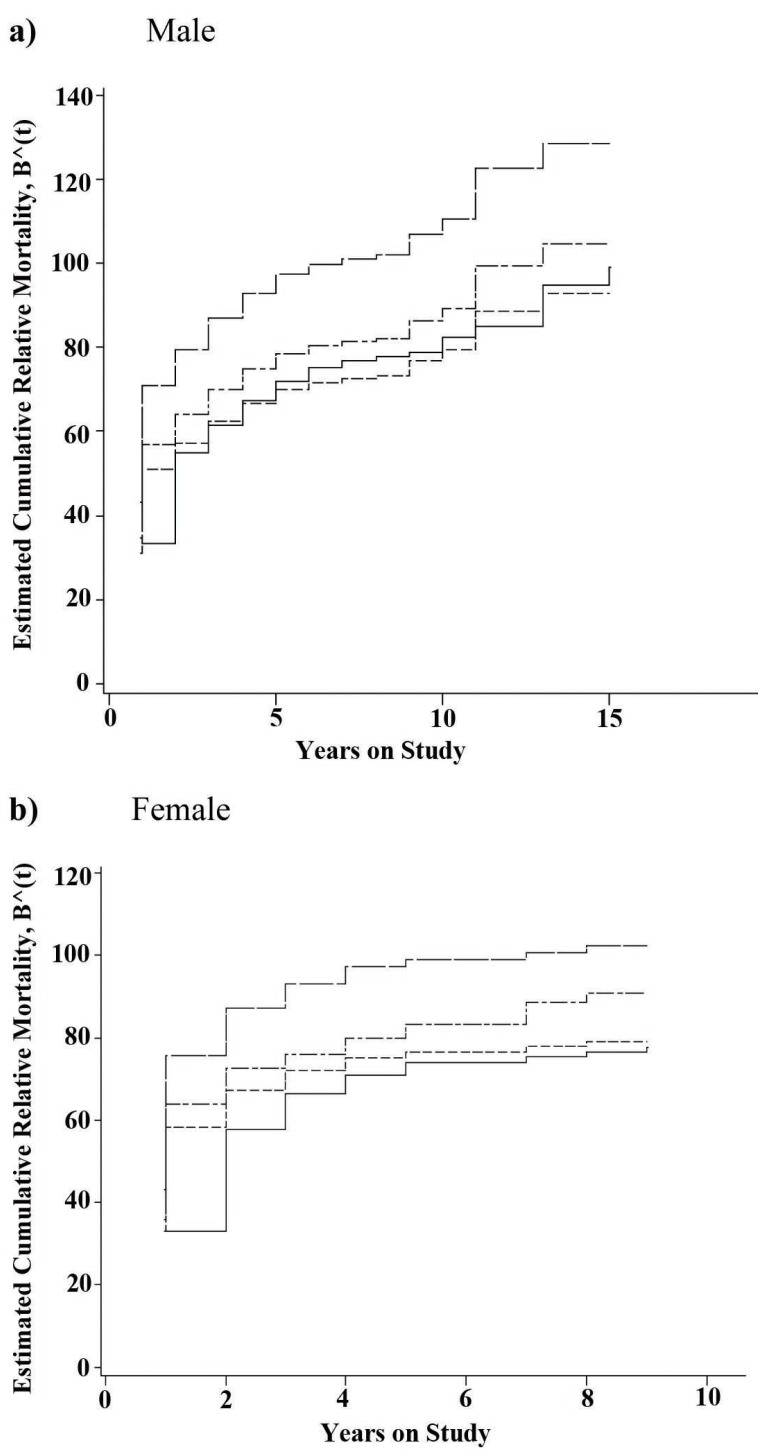
Estimated cumulative relative mortality for Mazandaran gastrointestinal tract cancer patients according to Coale- Demeny life table models. East (□ □ □), West (□ • □ • □), North (- - - - -), and South (solid line).

Figure 2 depicts the cumulative relative mortality for each gender according to the regional model life tables. We remind that a crude estimate of the relative risk is given by the slope of the estimated cumulative relative mortality. Therefore, it is clearly obvious that the pattern of change in relative mortality differs across the two genders over the course of study. In both genders, in the first two years of observation, patients are most likely to die than the reference population. In men, in years 3-10, the slope of cumulative relative mortality falls down slightly and increases gradually afterwards. In contrast, in women, the slope drops slowly during the years 3 and 4, and then levels off subsequently for the rest of the period. Of note, since the last death in women occurred 9 years after follow up, the estimates of the cumulative relative mortality was zero for the years 10-15 (Figure 2).

**Figure 3:**
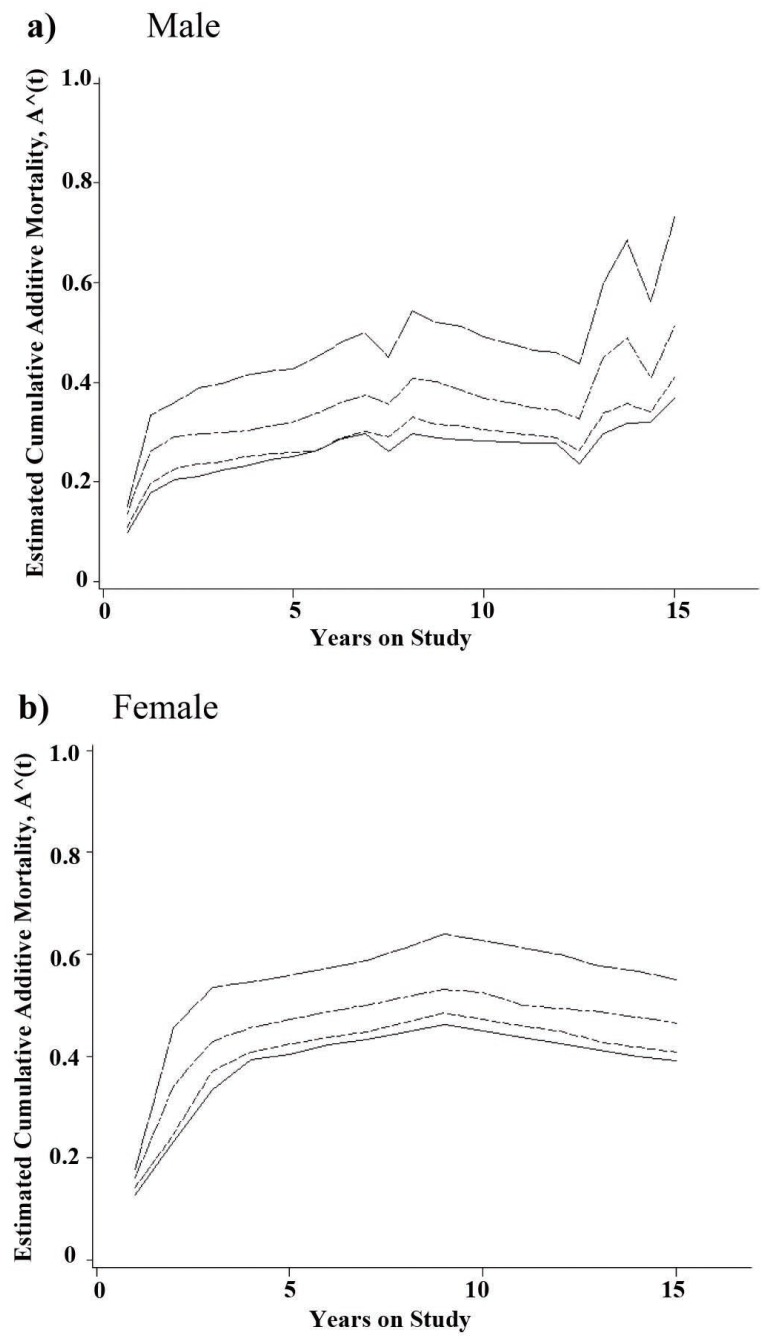
Estimated cumulative excess mortality for Mazandaran gastrointestinal tract cancer patients according to Coale-Demeny life table models. East (□ □ □), West (□ • □ • □), North (- - - - -), and South (solid line).

Regarding the cumulative excess mortality, it is also evident that the excess mortality pattern varies widely across the two genders (Figure 3). Again, in the first two years, the chance of dying of the cancer is considerably high for both genders when compared to the reference population. This chance reduced through the decline in the slope value by the years nearly nine and twelve, for women and men, respectively, and then climbed more quickly for the remaining period during which just men patients experienced the death event. It should be noted that the cumulative excess mortality has jumps at the death times and is decreasing between the death times (15). This is the reason for any reduction in cumulative additive mortality, especially for women in years 10 through 15 following cancer diagnosis (Figure 3).

It is interesting to point out that the estimates of both the relative and additive mortality reached the highest values over the course of study when using the East life table model to generate population mortality rates. The estimates reached the lowest values using the South model and they kept the middle values by the West life table model.

## DISCUSSION

In the present study, our effort was mainly focused on showing the effects of different life table patterns on the evaluation of the long-term excess mortality in a developing country in which registration systems are so affected by omission and other errors. To do so, first the Coale-Demeny life tables were established distinctly for each combination of birth cohort and sex of patients, as the information on the actual age pattern of mortality in our population was unreliable. Second, these established life tables were considered as the standard reference population in estimating excess mortality.

As was found, a considerable proportion of excess deaths occurred in the first two years of observation in both genders and when using each of the life table patterns. This might be expected because patients with GI tract cancers are generally diagnosed in a late stage of disease when cancer is also difficult to cure successfully at this stage (16, 17). For the years 3 to 10 after diagnosis, an alleviated excess mortality was observed. An explanation might be that patients diagnosed at a very late stage of cancer or those who were frail experienced death at an earlier time and those who survived longer after diagnosis had more chances of survival, although an excess mortality rate was still observed in these patients over the period. And for the years following the first decennial when the cancer diagnosed, excess mortality rate increased again in men with a steeper slope that could simply be described by the very small number of cases (about 9% of all men) survived a long time after cancer was detected. In other words, even though a relatively small number of deaths occurred (almost 10%) over the period, an overestimated excess mortality was obtained which could be considered an imprecise estimate.

The literature describing the relation between having a certain cancer and excess mortality is overwhelming, and in most of studies regression methods have been proposed as a general approach in describing the relation between cancer and excess hazard. Estimating excess hazard requires substituting the population mortality rate as a known value in the likelihood function. Not surprisingly, no adjustment has been usually made for birth cohort effect in constructing life tables (5, 6, 8, 15, 18-23). Sasieni proposed a proportional excess hazards model in estimating excess risk for 988 adult patients with non-Hodgkins lymphoma presentation, and population mortality rates from England and Wales were adjusted for cohorts by dividing patients to 5-year age groups and sex (24).

Another finding of our study is that the excess mortality reached the greatest values over the course of study when the East Coale-Demeny life table was used as the reference, and obtained the lowest values when using the South model and it always reached the middle values using the West life table model. These discrepancies in excess mortality estimates might be explained by the characteristics of the Coale-Demeny life tables. It may be briefly pointed as follows: The basis of the Coale-Demeny life table system is the mortality patterns exhibited in 192 actual life tables by sex. They selected these life tables from an original collection of 326 male and 326 female life tables. Analysis of 192 life tables showed four age patterns of mortality. These patterns were labeled North, South, East, and West. The North pattern is characterized by relatively low infant mortality, relatively high child mortality, and low mortality after age 50. The South pattern is characterized by high mortality under age 5 (particularly among infants), low adult mortality from age 40 to age 50, and high mortality over age 65. The East pattern exhibits relatively high infant mortality and high old-age mortality. And the West pattern is derived from the largest set of observed life tables [130] and is considered to represent the most general mortality pattern (11). Coale and Demeny recommended its use when reliable information is lacking for choosing one of the other patterns (10, 11, 25).

## LIMITATIONS

The current study is subject to a few limitations. We might better develop our understanding of excess mortality by performing computations separately for the three categories of cancer, but we would encounter the small sample size problem especially of the patients with colorectal cancer (n=15), which would preclude an accurate assessment of excess mortality over the course of follow up. Although the stage of GI cancer can seriously affect the time of death, depending on whether the cancer has spread outside the tract to nearby tissues, the stage of cancer was not determined at the time of data collection. Therefore, we were not able to stratify our analysis based on the stage of cancer and it appears our results here shows an average excess mortality for patients with GI cancers.

## CONCLUSION

First, in order to account for confounding effect of different mortality rates across different cohorts, adjustment for birth cohort effect might be highly recommended when establishing life tables. Second, in developing countries whose registration systems either do not exist or are so affected by omission and other errors, the Coale-Demeny life table models could be used in such cases. And third, the West model might be recommended over the Coale-Demeny models as a first choice to represent mortality patterns when reliable information is lacking for choosing one of the other patterns.
